# Biomedical Applications of Chitosan-Coated Gallium Iron Oxide Nanoparticles Ga_x_Fe_(3−x)_O_4_ with 0 ≤ x ≤ 1 for Magnetic Hyperthermia

**DOI:** 10.3390/molecules31010177

**Published:** 2026-01-02

**Authors:** Marta Orzechowska, Urszula Klekotka, Magdalena Czerniecka, Adam Tylicki, Dmytro Soloviov, Arkadiusz Miaskowski, Katarzyna Rećko

**Affiliations:** 1 Faculty of Physics, University of Bialystok, K. Ciołkowskiego 1L, 15-245 Białystok, Poland; k.recko@uwb.edu.pl; 2 Faculty of Chemistry, University of Bialystok, K. Ciołkowskiego 1K, 15-245 Białystok, Poland; u.klekotka@uwb.edu.pl; 3 Faculty of Biology, University of Bialystok, K. Ciołkowskiego 1J, 15-245 Białystok, Poland; m.siemieniuk@uwb.edu.pl (M.C.); atyl@uwb.edu.pl (A.T.); 4 European Molecular Biology Laboratory, Notkestraße 85, 22607 Hamburg, Germany; dimkaupml@gmail.com; 5 Faculty of Electrical Engineering and Computer Science, Lublin University of Technology, Nadbystrzycka 38A, 20-618 Lublin, Poland; a.miaskowski@pollub.pl

**Keywords:** magnetic nanoparticles, magnetic hyperthermia, heating efficiency

## Abstract

Nanoparticles based on gallium ferrite are explored as potential agents for magnetic fluid hyperthermia due to their magnetic performance and biocompatibility. In this study, Ga_x_Fe_3−x_O_4_ systems (0 ≤ x ≤ 1) were synthesized by co-precipitation of iron chlorides, with part of the series modified by a chitosan shell. Structural analysis confirmed single-phase formation across the studied range, while microscopy revealed irregular morphology, broad size distribution, and aggregation into mass-fractal-like assemblies. Chitosan was observed to coat groups of particles rather than single crystallites. Under an alternating magnetic field, all samples exhibited efficient heating, with specific absorption rate values generally increasing with gallium content. The composition Ga_0.73_Fe_2.27_O_4_ showed the highest SAR—83.4 ± 2.2 W/g at 2.8 mg/mL, 532 kHz, 15.3 kA/m, and SAR values rose with decreasing concentration. Cytotoxicity assays without magnetic activation indicated no harmful effect, while chitosan-coated nanoparticles enhanced fibroblast viability and lowered metabolic activity of HeLa cells. Higher Ga content (x = 0.66) combined with chitosan modification was identified as optimal for hyperthermia. The results demonstrate the biomedical potential of these nanoparticles, while emphasizing the need to reduce shape heterogeneity, aggregation, and sedimentation for improved performance.

## 1. Introduction

Magnetic nanoparticles (MNPs) are applied in many fields, including medicine, electronics and nanotechnology [1,2]. They form a broad family of materials, but in this work the focus is on ferrites, which typically preserve the crystal structure of their bulk counterparts [3]. For iron oxides, the spinel structure is retained, with Fe^2+^ and Fe^3+^ ions distributed between tetrahedral and octahedral sites [4,5,6,7]. This cation arrangement determines the magnetic properties. For example, the ferrimagnetic ordering in magnetite (Fe_3_O_4_) arises directly from the distribution of Fe ions in the spinel lattice [8,9]. Therefore, the crystal structure and exact metal-ion positions are essential for explaining and tailoring the magnetic behavior of ferrite nanoparticles [10]. In magnetite (Fd$\bar{3}$m), Fe ions can be partially substituted by dopant metal ions such as Ni^2+^, Mn^2+^, Mg^2+^, Zn^2+^, Al^3+^ and Ga^3+^, which occupy tetrahedral or octahedral sites within the crystal lattice, thus influencing the material’s physicochemical properties [11,12,13,14,15,16,17,18]. Due to their large surface-to-volume ratio, surface interactions play a particularly important role [19].

Magnetic nanoparticles can be coated with protective layers such as polymers, surfactants, carbon nanotubes or inorganic compounds like metal oxides to stabilize them and control their surface interactions [20,21]. An additional functionalizing layer often introduces specific chemical groups at the nanoparticle surface. Amino, carboxyl, thiol and hydroxyl groups enable dipolar interactions, hydrogen bonding and electrostatic interactions, affecting both stability and biological performance [22]. Among polymers, chitosan is widely studied because its amino and hydroxyl groups allow versatile surface modification and strong interfacial interactions, while its biocompatibility and biodegradability are crucial for biomedical applications [23]. For biological and medical uses, MNPs may be functionalized with biomolecules such as proteins, nucleic acids or polysaccharides (e.g., chitosan) [24,25,26,27]. The functionalizing layer is chosen according to the application: coatings for imaging diagnostics may contain contrast agents, whereas nanoparticles for oncological therapies may be functionalized with anticancer substances [28].

After many years of being overlooked, the doping of nanomagnetite with gallium has gained increasing importance in recent years [16,29,30,31]. The currently reported lethal dose for 50% of the population (LD_50_) for gallium, determined in rats, is 220 mg/kg [32]. For comparison, the human body contains approximately 40–50 mg of iron per kilogram of body weight [33]. Due to their physicochemical properties, gallium ferrites exhibit promising characteristics for potential biomedical applications [34]. The safety of gallium in medical applications depends primarily on its chemical form, dosage, route of administration, and duration of exposure. Gallium nitrate, which is used therapeutically, has a well-established safety profile and has been employed clinically to treat hypercalcemia of malignancy, lymphomas, and bladder cancer. Studies indicate that it is generally well tolerated, especially when administered via continuous infusion, which minimizes the risk of adverse effects. The most significant concern associated with therapy is nephrotoxicity, which is more likely to occur with rapid administration of high doses [35]. These observations reflect a broader trend towards developing gallium-based compounds with greater efficacy and, potentially, improved safety profiles compared with traditional gallium nitrate [36]. Hydroxyapatite with gallium nitrate and sodium gallate (0.05–5 mg/mL gallium content) shows no toxicity in CCL-81 monkey cells in vitro after 48 h, indicating that such materials may be characterized as biologically inert and compatible. These results open possibilities for application of gallium hydroxyapatite, for example, in orthopedic implants [37]. In contrast, gallium arsenide, widely used in electronics and photonics, poses entirely different risks due to its arsenic content and is therefore unsuitable for medical use, being restricted to industrial applications [35,38]. Other industrial materials, such as gallium phosphide nanowires, also show some toxic effects. Pulmonary exposure to these nanowires caused acute inflammation in the lungs of mice. The nanowires were observed to dissolve in vivo, which may reduce their long-term toxicity. Nevertheless, precautionary measures should be implemented to prevent potential human exposure during the production and post-production handling of semiconductor materials, including those containing gallium [39,40].

The toxic effects of gallium (GaCl_3_) on marine microorganisms (*Americamysis bahia*, *Brachionus plicatilis*, *Artemia salina*; LC_50_ between 10 and 50 mg/L) are significantly weaker than those of other metals such as copper, zinc and cadmium, and decrease with increasing salinity [41]. In swamp shrimp (*Macrobrachium nipponense*), iridium (21.5 mg/L) proved to be less toxic than gallium (12.7 mg/L), while other metals such as antimony, cadmium and copper showed significantly lower LC_50_ values (from 7.4 to 0.4 mg/L) [42]. Similarly, routine ecotoxicology tests in the tropical microalga *Isochrysis galbana* show high sensitivity to copper, while aluminum, gallium, and molybdenum exhibit little to no toxicity towards the species [43]. Gallium occurs in the Earth’s crust at very low concentration and is obtained as a by-product during the extraction of other metals. Given the limited availability of gallium in the natural environment, the lack of knowledge about metabolic reactions that strictly require gallium, as well as its relatively low toxicity compared with other metals, the incorporation of gallium admixture into ferrite magnetic nanoparticles appears safe and justified. This is particularly relevant considering its physical properties, especially in the context of magnetic hyperthermia.

The ionic radii of Ga^3+^ and Fe^3+^ are similar; therefore, interionic distances and lattice parameters are expected to remain unchanged regardless of the gallium doping level. The gallium ions are nonmagnetic, and by occupying iron positions they allow modifications to the magnetic exchange interactions [29].

Magnetic fluid hyperthermia (MFH) is the basis of a non-invasive cancer treatment method. The effect relies on raising the temperature inside the tumor to 41–50 °C, which induces apoptosis, or ultimately necrosis of the cells at 50 °C. This process is triggered by a series of metabolic reactions [44]. One method of introducing a suspension of magnetic nanoparticles dispersed in a biocompatible solvent into a tumor is direct injection. MNPs enter the cells through endocytosis, where they accumulate, and are subsequently heated under an external alternating magnetic field [45].

A magnetic field can penetrate tissue, allowing treatment of tumors in various areas of the patient’s body. The temperature increase during magnetic hyperthermia arises from the response of the particles to the alternating magnetic field. The heating time and rate are related to relaxation mechanisms known as Brownian and Néel relaxation [46]. In Néel relaxation, the magnetic moment rotates within a stationary nanoparticle, while in Brownian relaxation, the entire nanoparticle rotates, generating heat through friction. If exposure to the magnetic field exceeds the combined relaxation times, heat is produced through energy absorption. The heating efficiency depends on particle size, their response to the magnetic field, as well as the amplitude and frequency of the magnetic field. The natural resistance of the particles to the magnetic field leads to heat generation [47,48]. By increasing the temperature of the tumor microenvironment, magnetic fluid hyperthermia can be used to deliberately slow the growth of cancer cells or even eliminate them entirely. This method is less invasive compared to conventional cancer therapies such as chemotherapy or radiotherapy. Heating the tumor with magnetic fluid can be performed externally, which reduces the risk of complications associated with surgical procedures. Magnetic fluid hyperthermia enables precise therapy, as the location and intensity of heating can be controlled, thereby minimizing harmful effects on healthy tissues and organs surrounding the tumor. It can also be applied as an adjunct to other treatments, such as chemotherapy or radiotherapy, potentially increasing the sensitivity of cancer cells to conventional therapies and thus improving therapeutic outcomes. MFH offers treatment possibilities for patients with tumors that are difficult to access by other means, for example, due to their location deep inside the body or in proximity to vital anatomical structures. Compared with conventional cancer treatments, magnetic fluid hyperthermia can minimize side effects, as heating is largely confined to the tumor region rather than affecting the whole body [49,50,51].

In this study, single-phase gallium ferrite nanoparticles Ga_x_Fe_3−x_O_4_ with varying gallium content (hereinafter referred to as Ga_x_) were synthesized in both uncoated and chitosan-coated forms and systematically analyzed using the aforementioned methods. The aim was to correlate structural and morphological features with magnetic heating performance and biological response in order to identify the composition and surface modification most suitable for magnetic fluid hyperthermia applications. Throughout this work, chitosan-coated nanoparticles are referred to as chitosan@Ga_x_. The composition range 0 ≤ x ≤ 1 was selected because gallium can substitute iron across the entire spinel lattice without forming secondary phases, and this interval covers the region in which the strongest changes in magnetic anisotropy and heating behavior are expected.

## 2. Results

### 2.1. X-Ray Diffraction

**Figure 1:**
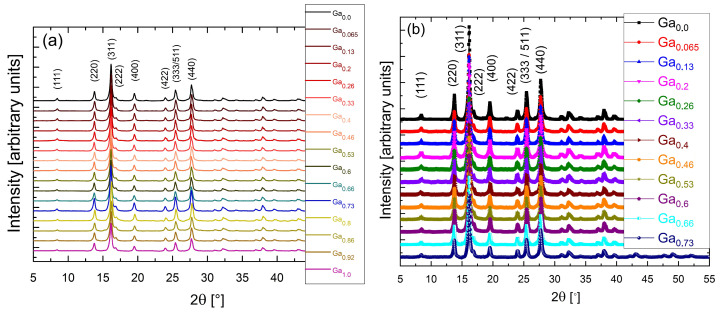
X-Ray diffraction patterns collected at room temperature: series without chitosan coating (**a**); series with chitosan coating (**b**).

Figure 1 presents the X-ray diffraction patterns of two series of nanoparticles: uncoated Ga_x_Fe_3−x_O_4_ (Figure 1a) and the corresponding samples coated with chitosan (Figure 1b).

In both series, all compositions exhibit a single-phase spinel structure, with diffraction peaks matching the standard pattern of cubic ferrite (ICSD Card No. 28282). No additional reflections were observed, confirming the high phase purity of the nanostructures. The most intense (311) reflection and the characteristic (333)/(511) doublet are clearly visible for all samples. As expected, the XRD patterns of the coated samples are identical to those of the uncoated series, as the chitosan layer is amorphous and does not contribute additional diffraction peaks. This confirms that the polymer shell does not modify the crystalline ferrite core. The broadening of the diffraction lines reflects the ultrafine nature and small crystallite size of the nanoparticles.

**Figure 2:**
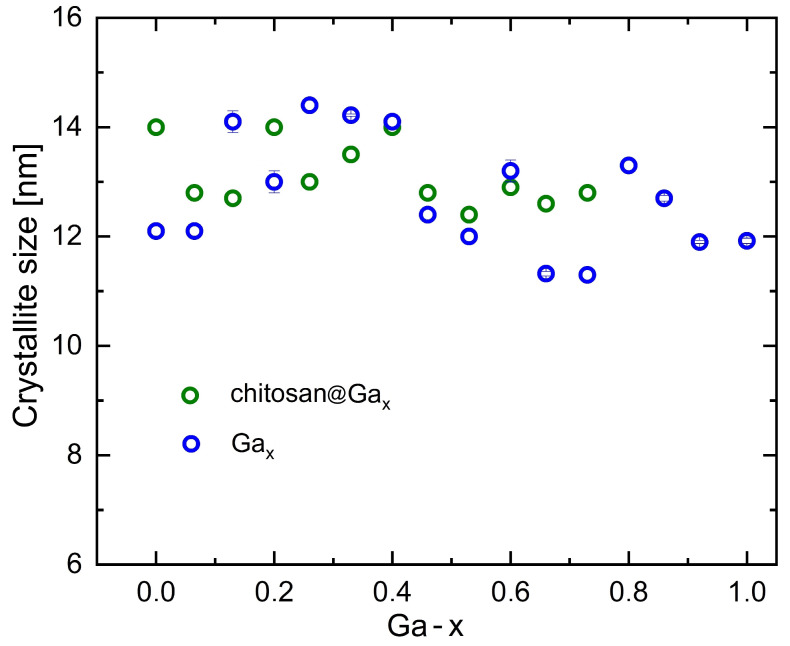
Crystallite size as a function of gallium content.

Figure 2 illustrates the crystallite size as a function of gallium content. The nanoparticle sizes range from 11 to 15 nm and decrease slightly with increasing gallium concentration, indicating that the co-precipitation method produces a relatively narrow size distribution. The crystallite sizes obtained from XRD reflect only the dimensions of the crystalline ferrite core. For this reason, the values for the coated and uncoated samples remain essentially the same, as the amorphous chitosan layer does not contribute to diffraction and therefore does not affect the calculated crystallite size. This behavior is fully consistent with previous reports showing that polymeric coatings, including chitosan, do not alter the crystalline structure of ferrite cores [52,53]. The gradual reduction in crystallite size with increasing Ga content is consistent with the known effect of gallium, which slows the crystal growth of spinel nanoparticles during precipitation.

### 2.2. Transmission Electron Microscopy Images

**Figure 3:**
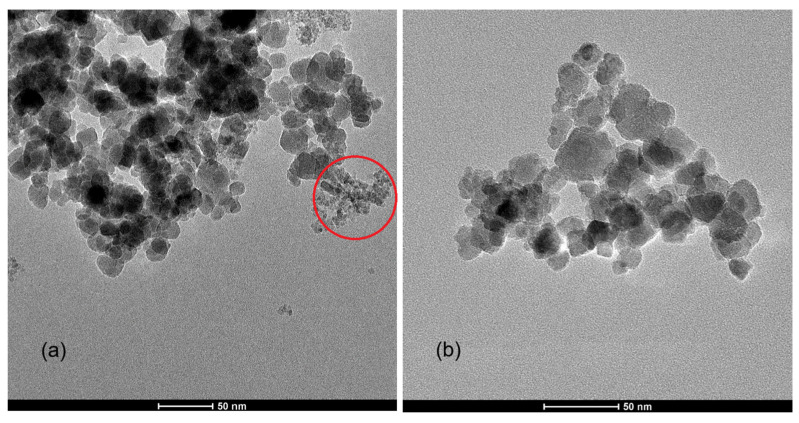
The TEM images of the nanostructures: Ga_0.33_ (**a**) and chitosan@Ga_0.6_ (**b**).

Below are TEM images of two gallium ferrite samples: Ga_0.33_ (Figure 3a) and chitosan@Ga_0.6_ (Figure 3b).

In the TEM image of Ga_0.33_ nanoparticles without the chitosan coating (Figure 3a), predominantly irregularly spherical and shape-heterogeneous particles are observed. The size distribution is relatively broad, indicating a polydisperse outcome of the synthesis process. A distinct tendency to form large aggregates is observed, which may result from magnetic interactions and van der Waals forces. In Figure 3a, areas free from aggregation are marked with a red circle, within which clear particle dispersion is visible. A structure similar to that typical of mass fractals is also observed.

The second TEM image shows Ga_0.6_ nanoparticles with the chitosan coating (Figure 3b). It was observed that, similar to the uncoated samples, these nanoparticles exhibit irregular shapes; however, their aggregation behavior differs significantly. The chitosan coating appears to influence the way the nanoparticles form aggregates, suggesting that chitosan does not surround individual nanoparticles but rather binds larger clusters, creating more compact and homogeneous structures. In an ideal scenario, where the polysaccharide surrounds individual nanoparticles, the chitosan coating should play a stabilizing role, reducing interactions between nanoparticles and inhibiting the formation of large aggregates.

These two compositions were chosen as representative examples of the uncoated and coated series. Because chitosan does not alter the crystalline core and is barely visible in TEM images, showing the same composition before and after coating would not provide additional structural information. Figure 3 is intended to illustrate the typical morphology of both series and the change in aggregation caused by the chitosan layer.

### 2.3. Small-Angle Neutron Scattering Measurements

To analyze the SANS (Small-Angle Neutron Scattering) data, two models were applied to describe nanoparticles of undefined shapes. The first model, GelFit, was used to describe the distribution of irregularly shaped nanoparticles, providing insights on particle size and their distribution. The mathematical expression for this model is [54]:

(1) $$I\left(Q\right)=\frac{I_{L}(0)}{{\left(1+\left[\frac{D+1}{3}\right]Q^{2}\zeta^{2}\right)}^{\frac{D}{2}}}+I_{G}\left(0\right)e^{\frac{-Q^{2}R_{g}^{2}}{3}}+background$$

where I(Q)—scattering intensity as a function of the scattering Q, $I_{L}(0)$—intensity coefficient of the Lorentzian contribution (related to particle size and fractal dimension), D—fractal dimension of the aggregates, ζ—cutoff length, $I_{G}(0)$—intensity coefficient of the Gaussian contribution (related to the internal structure of particles), and $R_{g}$—radius of gyration of the particles.

The second model applied was the mass fractal model, which is used for analyzing systems where particles form fractal-like structures, particularly relevant for nanoparticles with complex shapes. The formula for this model is [55]:

(2) $$\begin{array}{c} I\left(Q\right)=scale\times P\left(Q\right)S\left(Q\right)+background\\ P\left(Q\right)=F{\left(QR\right)}^{2}\;F\left(x\right)=3\frac{\sin\left(x\right)-x\cos\left(x\right)}{x^{3}}\\ S\left(Q\right)=\frac{\Gamma \left(D_{m}-1\right)\zeta^{D_{m}-1}}{{\left[1+{\left(Q\zeta \right)}^{2}\right]}^{\frac{D_{m}-1}{2}}}\frac{\sin\left[(D_{m}-1){tg}^{-1}(Q\zeta )\right]}{Q}\\ scale=scale_{factor}\times NV^{2}{\left(\rho_{particle}-\rho_{solvent}\right)}^{2}\;\;V=\frac{4}{3}\pi R^{3} \end{array}$$

where R—aggregate radius, $D_{m}$—mass fractal dimension, $\rho_{solvent}$—neutron scattering length density of the solvent, and $\rho_{particle}$—neutron scattering length density of the particles.

Both models were employed to describe the nanoparticles, enabling a more detailed understanding of their structure and properties.

**Figure 4:**
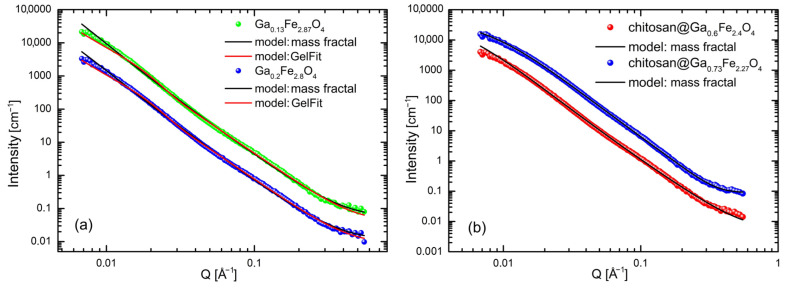
SANS data collected at 40 °C along with models describing selected gallium ferrite nanoparticles without the chitosan coating (**a**) and with the chitosan coating (**b**). Experimental data (symbols), model fit (solid lines).

Gallium ferrites, both uncoated and coated, did not exhibit a uniform shape. The I(Q) measurement series were well described by the mass fractal and GelFit models. Figure 4a,b present example measurements and fits for selected systems. The data shown were collected at a temperature of 40 °C.

The final data analysis was performed using the GelFit model, due to its best fit to the experimental results, which allowed for the determination of nanoparticle size. The results obtained at 40 °C, which included 146 points (npts), are summarized in Table 1.

### 2.4. Small-Angle X-Ray Scattering Analysis

The SAXS data were described using the unified Beaucage model, which combines a Guinier term and a power-law term with a structural cutoff [56,57]. This model allows scattering from finite-size objects and their fractal or surface organization to be accounted for. The following equation was used for fitting the I(Q) data:

(3) $$I\left(Q\right)={(B}_{1}Q^{-P_{1}}+G_{2})e^{\frac{-Q^{2}R_{2}^{2}}{3}}+B_{2}{\left(\frac{Q}{\mathrm{erf}\left(QR_{2}/\surd 6\right)}\right)}^{-P_{2}}+background$$

where Q—magnitude of the scattering vector ($Q=\frac{4\pi }{\lambda }\sin\frac{\theta }{2}$), $R_{2}$—radius of gyration $R_{g}$ corresponding to the given structural scale, $B_{1}$, $B_{2}$—prefactors of the power-law terms, $G_{2}$—prefactor of the Guinier term, $G=nV^{2}{\left(\bar{\rho }-\rho \right)}^{2}$ in the monodisperse approximation, and $P_{1}$ and $P_{2}$—power-law exponents (for 1 < P < 3—mass fractal D = P; for 3 < P < 4—surface fractal D_S_ = 6 − P).

**Figure 5:**
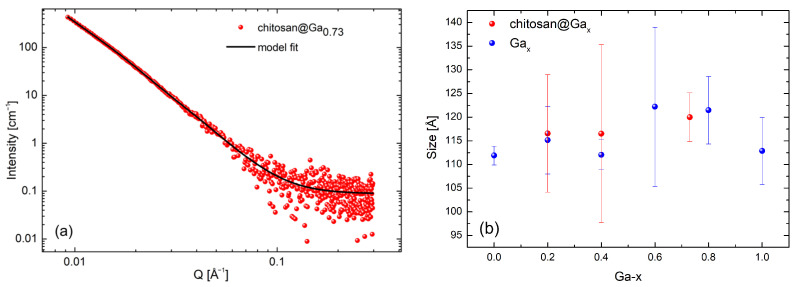
SAXS data with the model describing the system (**a**)—experimental data (symbols), model fit (solid lines), and nanoparticle sizes as a function of gallium concentration (**b**).

In Figure 5a, the SAXS data for system chitosan@Ga_0.73_ are shown together with the fit obtained using the above-described unified Beaucage model.

The nature of the obtained fits, as indicated by the values of the power-law exponents in the applied model, suggests a mass-fractal organization of the analyzed structures. Figure 5b shows the sizes of selected nanostructures with the obtained values ranging from 11 to 12 nm.

### 2.5. Characterization of the Specific Absorption Rate

The results were obtained for two measurement series, including gallium ferrites with and without a chitosan coating. In both series, an alternating magnetic field with parameters of f = 523 kHz and H = 15.3 kA/m was applied. Ferrifluids were tested at three different concentrations: 11.2 mg/mL, 5 mg/mL and 2.8 mg/mL in deionized water. Each measurement lasted about 900 s, with 700 s allocated for heating and 200 s for cooling. Each measurement was repeated three times. To improve nanoparticle dispersion, each ferrifluids sample was placed in an ultrasonic bath for several seconds before the measurement. Specific absorption rate (SAR) coefficients were systematically determined based on equation [46,58]:

(4) $$SAR=\frac{c}{\varphi }\frac{\Delta T}{\Delta t}=\frac{P}{m_{NMP}}$$

where c—specific heat capacity of the ferrofluid [$\frac{J}{{}^{\circ}Cml}$], φ—concentration of magnetic nanoparticles in the ferrofluid [mg/mL], $\frac{\Delta T}{\Delta t}$—rate of temperature change over time, P—power deposited by magnetic nanoparticles [W], and $m_{NMP}$—mass of magnetic nanoparticles in the ferrofluid [g].

**Figure 6:**
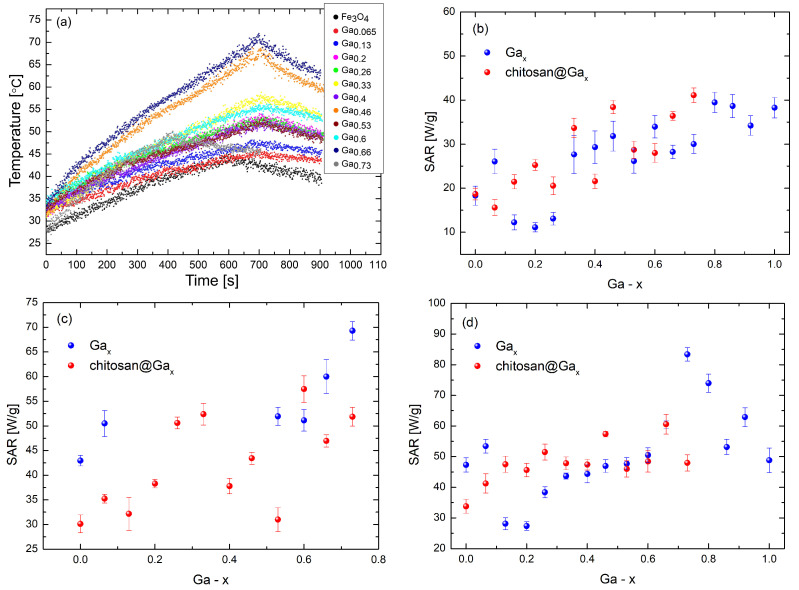
Heating characteristics of samples with a concentration of 11.2 mg/mL with a chitosan coating (**a**); variation in SAR with increasing gallium content for samples with concentrations of 11.2 mg/mL (**b**), 5 mg/mL (**c**), and 2.8 mg/mL (**d**).

The heating curves for the chitosan-coated series at a concentration of 11.2 mg/mL are presented in Figure 6a. The differences between the three tested concentrations arise from the collective behavior of the nanoparticles in dispersion. At the highest concentration (11.2 mg/mL), stronger dipolar interactions and partial blocking reduce the heating efficiency, whereas at 2.8 mg/mL the particles are more freely oscillating and therefore exhibit higher SAR values. The lower SAR observed in the chitosan-coated series is related to the increased viscosity around the particles and the restricted rotational motion caused by the polymer layer, which reduces the Brownian contribution to heating. The appearance of a SAR maximum near x ≈ 0.7 is consistent with the gallium-induced modification of magnetic anisotropy and the resulting optimization of the relaxation dynamics under the applied field. It should be noted that the position and magnitude of the SAR maximum depend on the nanoparticle concentration, which is why Figure 6b–d show different SAR peaks for the same gallium composition.

Figure 6b–d present the calorimetric measurement results for both series of analyzed gallium ferrite nanoparticles. In Figure 6d, it is evident that in the chitosan-coated series, the SAR coefficient increases systematically with the increasing gallium content, reaching the highest value of 60.6 ± 3.2 W/g for the chitosan@Ga_0.66_ sample. In the series without chitosan, the SAR coefficient begins to increase at x = 0.2, reaching the maximum value of 83.4 ± 2.2 W/g for the Ga_0.73_ sample.

Figure 6c shows fluctuating SAR values in the chitosan-coated nanoparticle series, with an overall increasing trend as the gallium concentration rises. The highest SAR coefficient in this series was 57.5 ± 2.7 W/g, observed for the chitosan@Ga_0.6_ composition. In the series without chitosan, the increasing trend became apparent at x = 0.53, with the highest SAR value of 69.3 ± 1.9 W/g achieved by the Ga_0.73_ system. For several compositions at this concentration, the temperature rise was too small to determine SAR reliably, which is why several data points in Figure 6b,d are not included in Figure 6c.

In Figure 6b, an increasing trend of the SAR coefficient is observed in both series—with and without chitosan—along with the increasing x-gallium content. For the gallium concentration range x = 0–0.73, the highest SAR coefficient of 41.1 ± 1.6 W/g was observed in the chitosan-coated series for the chitosan@Ga_0.73_ sample, while in the series without chitosan, the highest value of 34.0 ± 2.6 W/g was obtained for the Ga_0.6_ system. This value represents the highest SAR within the composition range (x = 0–0.73) common to both series, allowing a direct and consistent comparison between the coated and uncoated nanoparticles. Although the absolute maximum SAR for the uncoated series occurs at x = 0.8, this composition cannot be compared directly because the coated series does not extend beyond x = 0.73.

Based on the data presented in Figure 6b–d, it can be concluded that the most promising material for biomedical applications is Ga_0.73_Fe_2.27_O_4_. These nanoparticles exhibited the highest SAR coefficient in the entire measurement series at a concentration of 2.8 mg/mL. It is worth noting that 2.8 mg/mL is closest to typical clinical concentrations [59].

### 2.6. In Vitro Cytotoxicity Assessment

To evaluate the effect of the tested nanoparticles on normal cells (human skin fibroblasts) and cancer cells (HeLa cervical cancer cells) without magnetic activation, an in vitro experiment was conducted, during which the aforementioned cells were treated with nanoparticles at a concentration of 0.01 mg/mL. The tests were performed after three days of culture for HeLa cells and after seven days for fibroblasts, when the control culture reached approximately 90% confluence.

**Figure 7:**
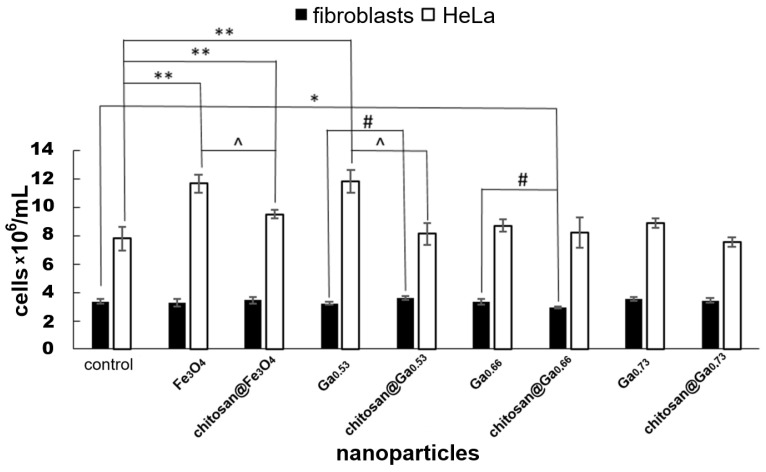
Comparison of the cell number in fibroblast and HeLa cultures exposed to the tested nanoparticles at a concentration of 0.01 mg/mL, results obtained after 7 days of culture in the case of fibroblasts and after 3 days of culture in the case of HeLa (data represent the mean ± SD; *—statistically significant difference compared to the control for fibroblasts; **—statistically significant difference compared to the control for HeLa cells; #—statistically significant differences between the corresponding pairs of chitosan-coated and uncoated nanoparticles in the case of fibroblasts; ^—statistically significant differences between the corresponding pairs of chitosan-coated and uncoated nanoparticles in the case of HeLa cells. ANOVA *p* ≤ 0.05, post hoc Tukey test *p* ≤ 0.05; SD—standard deviation).

In fibroblasts, nanoparticles did not significantly affect culture growth. A slight (13%) decrease in cell number relative to the control was observed only for the chitosan@Ga_0.66_ sample. The presence or absence of the chitosan coating also did not significantly influence the fibroblast growth. When comparing the effects of adequate nanoparticles without chtiosan coating to those with the coating, only minor fluctuations in cell number (approximately 11–13%) were observed (Figure 7).

In HeLa cultures increase in cell number (20–50%) was observed for nanoparticles without gallium and those with lower gallium content (0.53), while higher gallium content (0.66–0.73) did not affect HeLa cell number compared to control, regardless of the presence or absence of the chitosan coating. Interestingly, for HeLa cells exposed to nanoparticles without gallium or with low gallium content (0.53), presence of the chitosan coating led to a decrease in cell number (20–30%) compared to the corresponding cultures with uncoated nanoparticles. This trend, although not statistically significant, is also observed for nanoparticles with higher Ga content. In the case of fibroblasts only very slight decrease (about 10%) in cell numbers compared to the control were noted in the case of chitosan coated nanoparticles with 0.66 gallium content. Comparing the results obtained for nanoparticles with and without chitosan coating, we found a small, 12% increase in cell number in the case of lower gallium content (0.53) and an equally small decrease in cell number in the case of gallium content of 0.66 (Figure 7).

**Figure 8:**
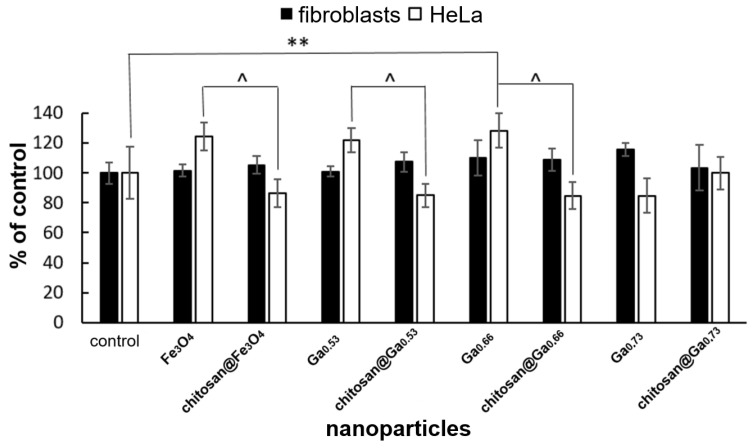
Cytotoxicity of nanoparticles in concentration of 0.01 mg/mL against fibroblasts and HeLa cells measured by MTT test (data represent % of control ± SD). Results obtained after 7 days of culture in the case of fibroblasts and after 3 days of culture in the case of HeLa. **—statistically significant difference compared to the control for HeLa cells; ^—statistically significant differences between the corresponding pairs of chitosan-coated and uncoated nanoparticles in the case of HeLa cells; ANOVA *p* ≤ 0.01, post hoc Tukey test *p* ≤ 0.01.

A similar trend was observed in the cytotoxicity of nanoparticles against HeLa cells measured by MTT test (Figure 8). Chitosan coated nanoparticles without gallium as well as with gallium 0.53 and 0.66 proved to be more cytotoxic (approximately 30%) compared to adequate nanoparticles without chitosan coating. At the same time, no cytotoxic influence of nanoparticles on fibroblasts were observed, regardless of the presence or absence of the chitosan coating.

**Figure 9:**
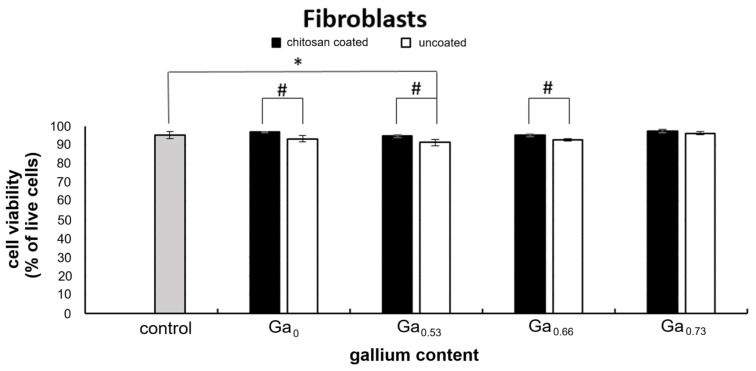
Comparison of cell viability (express as % of live cells) in control fibroblast cultures and those exposed to the nanoparticles at a concentration of 0.01 mg/mL of medium. Results obtained after 7 days of culture. *—statistically significant difference compared with the control; #—statistically significant differences between the corresponding pairs of chitosan-coated and uncoated nanoparticles. ANOVA *p* ≤ 0.01, post hoc Tukey test *p* ≤ 0.01.

Analysis of the cell-viability data revealed a slight cytoprotective effect of the chitosan-coated nanoparticles in fibroblast cultures. Cultures exposed to chitosan coated nanoparticles without gallium and those with 0.53 and 0.66 gallium addition showed higher viability compared with the corresponding cultures with uncoated nanoparticles. The strongest cytoprotective effect was observed for nanoparticles Fe_3_O_4_ (over 50% fewer dead cells). As the gallium content in the nanoparticles increased, a reduction in the observed effect was noted (Figure 9).

**Figure 10:**
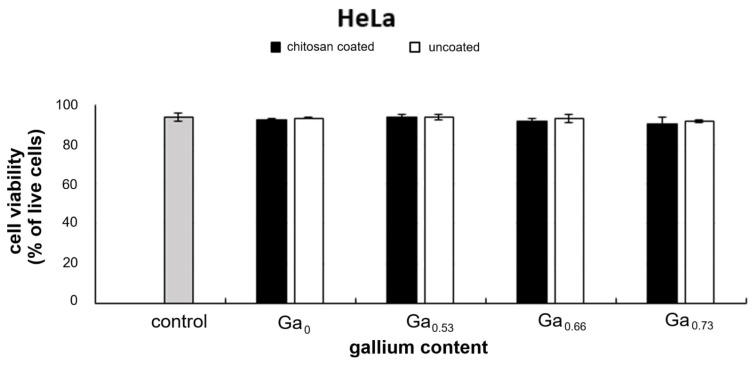
Comparison cell viability (express as % of live cells) in control HeLa cultures and those exposed to the tested nanoparticles at a concentration of 0.01 mg/mL. Results obtained after 3 days of culture. No statistically significant differences were found (ANOVA *p* ≥ 0.01).

In contrast no significant changes in HeLa cells viability were detected under the influence of the tested nanoparticles, regardless of the gallium content or the presence or absence of the chitosan coating (Figure 10).

## 3. Discussion

Diffraction measurements confirmed that the synthesized Ga_x_Fe_3−x_O_4_ nanoparticles were single-phase across the composition range Ga_x_ = 0–1. Transmission electron microscopy images revealed irregular particle morphology, a broad size distribution and high polydispersity, with a tendency to form large aggregates and mass-fractal-like structures.

The chitosan coating did not encapsulate individual nanoparticles but instead sur-rounded clusters, contrary to the expected stabilizing effect. This observation is highly relevant given that solubility, colloidal stability, and aggregation behavior of magnetic nanoparticles are among the key parameters determining their suitability for biological applications, including magnetic hyperthermia. Nanoparticles that remain stable under quiescent conditions may undergo pronounced aggregation when subjected to an alternating magnetic field [60]. Magnetically induced aggregation negatively affects magnetothermal performance, typically reducing SAR values, and even strong electrostatic stabilization does not fully prevent this process. Although interventions such as sonication may temporarily restore colloidal stability, the phenomenon remains a major limitation. Aggregation also has serious consequences in vivo: large clusters (>200 nm) exhibit reduced vascular permeability and compromised tissue distribution, which markedly lowers therapeutic effectiveness [61,62]. Furthermore, aggregation may alter the intrinsic magnetic properties of nanoparticles. Despite these drawbacks, aggregation is not always detrimental. Controlled intracellular aggregation triggered by tumor-specific conditions such as lowered pH has been shown to enhance nanoparticle retention within tumor tissue and increase heating efficiency [63]. Such aggregation strengthens local hyperthermia and modulates immune responses, including macrophage polarization and T-cell recruitment. However, under typical physio-logical conditions, aggregation remains undesirable and should be minimized using appropriate surface coatings, environmental control, and nanoparticle modification strategies. In the present study, SANS analysis indicated that the GaxFe_3−x_O_4_ nanoparticles can be described by a shape-independent Gel-Fit model, while SAXS confirmed their mass-fractal character. All samples heated efficiently under an alternating magnetic field. In both series, SAR values exhibited a general increase with gallium content, with no clear correlation to the presence of chitosan. The composition Ga_0.73_ displayed the highest SAR value of 83.4 ± 2.2 W/g at a concentration of 2.8 mg/mL (f = 532 kHz, H = 15.3 kA/m). For this composition, SAR values increased as the sample concentration decreased, and at the lowest concentration also rose with frequency. These results are consistent with previous studies on Ga_x_Fe_3−x_O_4_ nanoparticles for magnetic hyperthermia, where similar SAR ranges were reported [64], allowing for a quantitative comparison that highlights the influence of gallium content on heating efficiency. Our earlier research [34] revealed that the highest heat capacity typical for Fe_3_O_4_@Ga_0.6_Fe_2.4_O_4_ and Ga_0.6_Fe_2.4_O_4_@Fe_3_O_4_ nanoparticles is accompanied by a slight stimulation of fibroblast culture growth and inhibition of HeLa cell growth. In vitro cytotoxicity tests performed in the present study showed that gallium-doped nanoparticles, both chitosan-coated and uncoated exhibited only a slight effect on the growth of the tested cell lines in the absence of magnetic activation. The chitosan coating exerted a modest cytoprotective effect on human skin fibroblasts (reflected in slight increase in cell viability) and reduction in metabolic activity in HeLa cells compared with adequate gallium content uncoated nanoparticles. There is no additional literature on the effects of gallium-doped nanoparticles in in vitro models. However, there are data about other compositions of iron nanoparticles. For example, nickel-enriched iron nanoparticles (NiFe_2_O_4_) also exhibit low cytotoxicity in a concentration range from 1 to 100 μg/mL with regard to HeLa and PC-3 cancer cells [65]. Same results were also observed for cobalt-contained iron nanoparticles (CoFe_2_O_4_) on rat kidney proximal tubular epithelial cells (CRL-1571). They did not affect the cell viability at concentration ranging from 100 to 1000 µg/mL, but significantly induced DNA damage [66]. On the other hand, cobalt ferrite nanoparticles showed growth and viability inhibition as well as induction of apoptosis in experimental model of acute myeloid leukemia (CML) K562 cell line [67]. Manganese ferrite (MnFe_2_O_4_) magnetic nanoparticles showed dose dependent cytotoxic effect against 4T1murine breast cancer cell line. IC_50_ (half maximal inhibitory concentration) values was established at the range of 171–210 µg/mL after 24–72 h of incubation. Authors also noticed that at higher concentrations apoptosis and necrosis increased markedly, whereas concentrations below 125 µg/mL did not affect tested cancer cell line [68]. Cytotoxic potential was also established for copper ferrite nanoparticles in human lung (A549) and liver (HepG2) cells. Lung cells showed higher susceptibility than liver cells. Authors observed induction of reactive oxygen species after nanoparticles treatment which correspond to loss of mitochondrial membrane potential. In signs of apoptosis activation was also observed [69]. Considering the above facts, it can be concluded that the impact of ferrite nanoparticles enriched with various metals varies depending on the toxicity of the additives. When considering the toxicity of nanoparticles containing iron and copper, the possibility of activating the Fenton reaction and the Haber-Weiss reaction, which can generate particularly toxic hydroxyl radicals, should be taken into account.

In the case of gallium-containing nanoparticles presented in our work, the toxic effect on normal cells (fibroblasts) is marginal. In addition, traces of the cytoprotective effect of the chitosan shell are noticeable. Cancer cells, on the other hand, respond to chitosan with a slight reduction in metabolic rate. Considering the proposed use of these nanoparticles in magnetically induced thermotherapy, the absence of significant cytotoxic effects is advantageous. Further research should focus on methods of targeting these nanoparticles to cancer sites. The chitosan shell, which provides reactive chemical groups, offers possibilities for chemical modification of nanoparticles for this purpose.

In summary, Ga_x_Fe_3−x_O_4_ nanoparticles produced via the co-precipitation of iron chlorides exhibit high oncotherapeutic potential, particularly for 0.6 ≤ x ≤ 0.73. Chitosan modification improves their biological performance, although full optimization would benefit from a standardized synthesis procedure. Remaining challenges include particle shape heterogeneity, aggregation, and sedimentation in ferrofluid suspensions. The results presented for our ferrite nanoparticles with gallium addition in confrontation with literature data confirm that the observed SAR trends and biological responses show their suitability for hyperthermia applications.

## 4. Materials and Methods

The co-precipitation of chlorides (Massart method) relies on the precipitation of iron salts in the presence of a base [70]. The method offers short reaction times, mild conditions, and scalability, but it also tends to produce broad particle size distributions, limited reproducibility, and surfaces prone to oxidation [71,72]. Gallium ferrite nanoparticles synthesized using this approach were used in the present study.

Two types of materials were prepared: Ga_x_Fe_3−x_O_4_ cores (Ga_x_: x = 0–1) and chitosan-coated cores (chitosan@Ga_x_: x = 0–0.73). Chitosan, derived from chitin, is biocompatible and non-toxic, which enables its application in magnetic hyperthermia, imaging and drug-delivery systems [24,25,26,27,73,74,75,76].

The synthesis was performed under an argon atmosphere. The inert gas minimized Fe^2+^ oxidation during precipitation and prevented the formation of maghemite as a secondary phase. A solution of ammonia and TMAOH (tetramethylammonium hydroxide) was used as the reaction medium. FeCl_3_ was placed in flask A, while a mixture of FeCl_2_ and Ga(NO_3_)_3_ was placed in flask B. After heating both flasks to 40 °C for 20 min, the solutions were combined, heated to 80 °C for 30 min, and the resulting particles were magnetically separated. The product was washed with acetone for 3 days and dried in a rotary evaporator. All reagents were purchased from Sigma-Aldrich (St. Louis, MO, USA) and used without further purification. As an example, the Ga_0_._73_Fe_2_._27_O_4_ composition was synthesized from FeCl_3_ (0.528 g), FeCl_2_ (0.348 g), and Ga(NO_3_)_3_ (0.281 g). For the remaining compositions, precursor masses were adjusted stoichiometrically according to the intended gallium content.

The chitosan coating was obtained by dispersing nanoparticles in a TBAOH (tetra-n-butylammonium hydroxide) solution and adding a chitosan solution in acetic acid dropwise. After 1 h of mixing at room temperature, the particles were washed with ethanol and deionized water and dried overnight at 60 °C.

A comprehensive characterization of the systems required complementary methods. X-ray diffraction (XRD) provided information on crystal structure and phase composition, while transmission electron microscopy (TEM) allowed identification of particle morphology. Small-angle neutron scattering (SANS) and small-angle X-ray scattering (SAXS) were used to analyze size distribution, aggregation state and mass-fractal structures. These techniques probe the nanoparticles directly in dispersion, providing information not accessible by XRD or TEM. SANS and SAXS enabled verification of the core–shell organization, approximate estimation of the chitosan-layer thickness, and assessment of whether the coating affects nanoparticle clustering. Together, these data complemented the structural and morphological characterization and clarified the behavior of the systems under application-relevant conditions. Magnetic heating was evaluated through SAR measurements performed under strictly defined alternating-field conditions, while in vitro cytotoxicity assays assessed biocompatibility and potential therapeutic selectivity.

### 4.1. X-Ray Diffraction

XRD measurements were performed at room temperature using an Empyrean PANalytical diffractometer (Faculty of Physics, University of Bialystok). The instrument operated at 40 kV and 40 mA with MoKα radiation (λ = 0.7093187 Å) in Bragg–Brentano geometry. Diffraction patterns were collected using a PixCel1D linear detector over a 2θ range of 5–55° with a step size of Δ2θ = 0.026°. Instrumental broadening was corrected using the NIST LaB_6_ (660c) standard. Data were analyzed using Rietveld refinement (FullProf) [77], and phases were identified with the ICSD database [78] in the HighScore 4.0 package [79].

### 4.2. Transmission Electron Microscopy Images

The morphology and microstructure of the particles were examined using TEM at the Center for Synthesis and BioNanoTechno Analytical Methods, Faculty of Chemistry, University of Bialystok. Observations were performed with an FEI Tecnai G2 electron microscope operated at an accelerating voltage of 200 kV.

### 4.3. Small-Angle Neutron Scattering Measurements

SANS data were acquired using the YuMO spectrometer installed at the IBR-2 reactor. Measurements for each sample were performed at four temperature points within the range of 20–50 °C. Particle characteristics, including shape, size, morphology, and dispersion parameters were analyzed using SAS-view 5.0.3 software [80].

### 4.4. Small-Angle X-Ray Scattering Analysis

SAXS measurements were carried out at the Institute of Physics, Adam Mickiewicz University in Poznań, using a NanoSTAR system (Bruker-AXS). The instrument was equipped with a Cu X-ray source operating at 1.5 kW, crossed Göbel mirrors, a pin-hole collimator (0.1 mm and 0.3 mm apertures), and a two-dimensional gas detector PPG HI-STAR. The accessible size range for the measurements was from 10 Å to 1000 Å.

### 4.5. Characterization of the Specific Absorption Rate

The specific absorption rate (SAR) was determined from calorimetric measurements performed under non-adiabatic conditions using a magneTherm system (nanoTherics, Staffordshire, UK). Data acquisition and processing were carried out using the corrected slope method (CSM) [46,58]. Samples were placed in 3 mL Eppendorf tubes containing 2 mL of ferrofluid and positioned inside a 100.5 mL polystyrene foam jacket forming part of the measurement setup. Two series of materials were examined: gallium ferrites without coating and gallium ferrites coated with chitosan. In both cases, an alternating magnetic field with a frequency of 523 kHz and an intensity of 15.3 kA/m was applied. Measurements were conducted for three suspension concentrations in deionized water: 11.2 mg/mL, 5 mg/mL, and 2.8 mg/mL.

### 4.6. In Vitro Cytotoxicity Assessment

In vitro cytotoxicity assays were performed at the Faculty of Biology, University of Bialystok. Fibroblasts (ATCC-CRL-2106) and HeLa cells (ATCC-CCL-2) were maintained in MEM medium (M4665, Sigma-Aldrich) supplemented with 10% fetal bovine serum (F7524, Sigma-Aldrich), penicillin (50 U/mL) and streptomycin (50 µg/mL) (P0781, Sigma-Aldrich) under a humidified atmosphere of 5% CO_2_ at 37 °C in a NUAIRE NU-5820E incubator. Cells were seeded at a density of 1 × 10^5^ cells/well in 12-well plates and incubated overnight to allow attachment.

Control cultures (without nanoparticles) and experimental cultures (0.1, 0.05, 0.01 mg/mL) were grown until control wells reached 95% confluence (7 days of in the case of fibroblasts and 3 days in the case of HeLa). Nanoparticles were sterilized in 70% ethanol for 30 min, dried at 60 °C overnight, and dispersed in culture medium using a Hielscher UP50H ultrasonic homogenizer (cycle 0.5 s, amplitude 100%, 60 s + 3 min on ice).

Cell viability was determined using an EVE™ cell counter (NanoEnTec Inc., Seoul, Korea) with 0.2% trypan blue staining [81]. Cytotoxicity was assessed using the MTT assay [82] (M2128, Sigma-Aldrich) in PBS (5 mg/mL, 30 min), followed by dissolution of formazan crystals in 0.5 mL DMSO with 0.01 mL Sorensen’s buffer (0.1 M glycine, 0.1 M NaCl, pH 10.5). Absorbance was measured at 570 nm using a Lambda E plate reader (MWG AG BIOTECH).

Data from six replicates were analyzed using the Shapiro–Wilk test for normality and Levene’s test for homogeneity of variance, followed by one-way ANOVA with Tukey’s post hoc test (*p* ≤ 0.01). Results were expressed as mean ± standard deviation, and cell viability was reported as the percentage of live cells. Statistical analysis was performed using STATISTICA 13.3 (StatSoft Inc., Tulsa, OK, USA).

## Figures and Tables

**Table 1:** Results of the SANS measurements collected at 40 °C described by the GelFit model.

Sample Without Chitosan	Nanoparticle Size [nm]	$\frac{\chi^{2}}{npts}$	Sample with Chitosan	Nanoparticle Size [nm]	$\frac{\chi^{2}}{npts}$
Fe_3_O_4_	8.87 ± 0.16	1.5	Fe_3_O_4_	9.36 ± 0.18	1.1
Ga_0.065_	10.25 ± 0.10	1.4	Ga_0.065_	10.28 ± 0.12	1.8
Ga_0.13_	13.50 ± 0.11	4.7	Ga_0.13_	10.56 ± 0.13	1.6
Ga_0.2_	12.71 ± 0.11	4.4	Ga_0.2_	10.68 ± 0.11	2.0
Ga_0.26_	11.64 ± 0.20	1.6	Ga_0.26_	10.32 ± 0.17	2.1
Ga_0.33_	10.89 ± 0.13	1.6	Ga_0.33_	10.36 ± 0.22	2.2
Ga_0.4_	11.20 ± 0.17	1.4	Ga_0.4_	10.27 ± 0.12	2.5
Ga_0.46_	10.67 ± 0.15	1.4	Ga_0.46_	10.37 ± 0.20	1.4
Ga_0.53_	10.48 ± 0.18	1.7	Ga_0.53_	10.47 ± 0.12	1.9
Ga_0.6_	11.11 ± 0.13	1.8	Ga_0.6_	11.40 ± 0.12	2.7
Ga_0.66_	10.20 ± 0.16	1.1	Ga_0.66_	9.60 ± 0.13	1.4
Ga_0.73_	8.86 ± 0.12	1.4	Ga_0.73_	10.17 ± 0.20	2.3
Ga_0.8_	10.32 ± 0.18	1.4			
Ga_0.86_	10.49 ± 0.16	1.2			
Ga_0.92_	10.02 ± 0.13	1.4			
Ga_1.0_	10.14 ± 0.12	1.0			

## Data Availability

The original contributions presented in this study are included in the article. Further inquiries can be directed to the corresponding author.
